# Introduction to metallic nanoparticles

**DOI:** 10.4103/0975-7406.72127

**Published:** 2010

**Authors:** Vicky V. Mody, Rodney Siwale, Ajay Singh, Hardik R. Mody

**Affiliations:** Department of Pharmaceutical Sciences, Appalachian College of Pharmacy, 1060 Dragon Road, Oakwood, Virginia USA 246 14; 1 Department of Radiology, UT Southwestern Medical Center, 5323 Harry Hines Boulevard, Dallas, Texas 753 90, India; 2 Department of Radiology, Dr. L.H. Hiranandani College of Pharmacy, Mumbai University, Ulhasnagar-421 003, India

**Keywords:** Fe_3_O_4_, gold nanoparticles, iron oxide nanoparticles, metallic nanoparticles, nanocages, nanoshells, silver nanoparticles

## Abstract

Metallic nanoparticles have fascinated scientist for over a century and are now heavily utilized in biomedical sciences and engineering. They are a focus of interest because of their huge potential in nanotechnology. Today these materials can be synthesized and modified with various chemical functional groups which allow them to be conjugated with antibodies, ligands, and drugs of interest and thus opening a wide range of potential applications in biotechnology, magnetic separation, and preconcentration of target analytes, targeted drug delivery, and vehicles for gene and drug delivery and more importantly diagnostic imaging. Moreover, various imaging modalities have been developed over the period of time such as MRI, CT, PET, ultrasound, SERS, and optical imaging as an aid to image various disease states. These imaging modalities differ in both techniques and instrumentation and more importantly require a contrast agent with unique physiochemical properties. This led to the invention of various nanoparticulated contrast agent such as magnetic nanoparticles (Fe_3_O_4_), gold, and silver nanoparticles for their application in these imaging modalities. In addition, to use various imaging techniques in tandem newer multifunctional nanoshells and nanocages have been developed. Thus in this review article, we aim to provide an introduction to magnetic nanoparticles (Fe_3_O_4_), gold nanoparticles, nanoshells and nanocages, and silver nanoparticles followed by their synthesis, physiochemical properties, and citing some recent applications in the diagnostic imaging and therapy of cancer.

Nanotechnology refers to the branch of science and engineering dedicated to materials, having dimensions in the order of 100th of nm or less.[1] The term being new, but has been widely used for the development of more efficient technology. In recent years, nanotechnology has been embraced by industrial sectors due to its applications in the field of electronic storage systems,[2] biotechnology,[3] magnetic separation and preconcentration of target analytes, targeted drug delivery,[4,5] and vehicles for gene and drug delivery.[2,4–6] Consequently, with wide range of applications available, these particles have potential to make a significant impact to the society. Although new, the history of nanomaterials dates long back to 1959, when Richard P. Feynman, a physicist at Cal Tech, forecasted the advent of nanomaterials. In one of his class he said, “There is plenty of room at the bottom,” and suggested that scaling down to nanolevel and starting from the bottom was the key to future technology and advancement.[6] As the field of nanotechnology advanced, novel nanomaterials become apparent having different properties as compared to their larger counterparts. This difference in the physiochemical properties of nanomaterials can be attributed to their high surface-to-volume ratio. Due to these unique properties, they make excellent candidate for biomedical applications as variety of biological processes occur at nanometer scales.

In general, nanoparticles used in the field of biotechnology range in particle size between 10 and 500 nm, seldom exceeding 700 nm. The nanosize of these particles allows various communications with biomolecules on the cell surfaces and within the cells in way that can be decoded and designated to various biochemical and physiochemical properties of these cells.[7] Similarly, its potential application in drug delivery system and noninvasive imaging offered various advantages over conventional pharmaceutical agents.[7] In an effort to utilize nanoparticles at their full throttle, it is important that the nanoparticulate systems should be stable, biocompatible, and selectively directed to specific sites in the body after systemic administration. More specific targeting systems are designed to recognize the targeted cells such as cancer cells. This can be achieved by conjugating the nanoparticle with an appropriate ligand, which has a specific binding activity with respect to the target cells. In addition, nanoparticles provide a platform to attach multiple copies of therapeutic substance on it and hence increase the concentration of therapeutic and diagnostic substances at the pathological site. Also, the concentration and dynamics of the active molecule can be varied by controlling the particle size of nanoparticles (>3–5 nm). This control in particle size in conjugation with surface coating with stealth ligand allows them to veil against body’s immune system, enabling them to circulate in the blood for longer period of time.[7] These advances in the field of biotechnology have opened an endless opportunities for molecular diagnostics and therapy.[8] Once targeted (active or passive), these nanocarriers can be designed in a way to facilitate them to act as imaging probes using variety to techniques such as ultrasound (US), X-ray, computed tomography (CT), positron emission tomography (PET), magnetic resonance imaging (MRI), optical imaging, and surface-enhanced Raman imaging (SERS) [Table 1].[9] Hence, these so-called “molecular imaging probes” can noninvasively provide valuable information about differentiate abnormalities in various body structures and organs to determine the extent of disease, and evaluate the effectiveness of treatment.[7] Thus short molecular imaging enables the visualization of the cellular function and the follow-up of the molecular process in living organisms without perturbing them.[10]

**Table 1:** Comparison of common imaging techniques along with the nanoparticles currently used or under clinical trials[10]

Technique	Advantages	Disadvantages	Nanoparticles used
Ultrasound	Easy to perform	Resolution of images is often limited	Fe_2_O_3_, Gd_2_O_3_
	Noninvasive	Reflected very strongly on passing from tissue to gas, or vice versa	
	No radiation hazard because it has nonionizing radiation being emitted	Does not pass well through bone	
	Relatively inexpensive as compared to the other imaging modalities currently available	Attenuation can reduce the resolution of the image	
CT	Wide field of view,	Need for contrast agents for enhanced soft tissue contrast	Gold and silver nanoparticles
	Detection of even subtle differences between body tissues	Radiations	Multimodal imaging nanoparticles
	Ability to provide cross sectional images of the body	Tissue non-specificity	
		Cost	
PET	Can image biochemical and physiological phenomena	Radiations	Radioactive ^64^Cu, ^62^Cu, ^82^Rb, and ^68^Ga, with particles tagged or conjugated with any organic moiety containing ^19^F being with the first choice
		Some tumors show poor FDG affinity	
		FDG uptake is not limited to the tumor cells and is prevalent in other benign cells	
		Motion artifact is the serious problems	
		Resolution of images is lower as compared to CT or MRI. This results in poor localization of lesions	
		Interpretation is very challenging	
		Most expensive technique	
MRI	Higher resolution	Expensive to use	Iron oxide nanoparticles are most commonly used, but newer generation multimodal imaging agents are also considered for clinical trials
	Can show the anatomical details	Cannot be used in patients with metallic devices, like pacemakers	
	Does not use any ionizing radiation		

Over the year’s nanoparticles such as magnetic nanoparticles (iron oxide), gold and silver nanoparticles, nanoshells, and nanocages have been continuously used and modified to enable their use as a diagnostic and therapeutic agent. Thus, in this particular review article we have introduced iron oxide, gold, and silver nanoparticles along with newer nanoshells and nanocages. These are then briefly discussed for their method of development and some citing recent examples which utilize their intrinsic properties as diagnostic and/or therapeutic agents for diseases, mainly cancer.

## Iron Oxide Nanoparticles

Iron (III) oxide (Fe_2_O_3_) is a reddish brown, inorganic compound which is paramagnetic in nature and also one of the three main oxides of iron, while other two being FeO and Fe_3_O_4_. The Fe_3_O_4_, which also occurs naturally as the mineral magnetite, is also superparamagnetic in nature. Due to their ultrafine size, magnetic properties, and biocompatibility, superparamagnetic iron oxide nanoparticles (SPION) have emerged as promising candidates for various biomedical applications, such as enhanced resolution contrast agents for MRI, targeted drug delivery and imaging, hyperthermia, gene therapy, stem cell tracking, molecular/cellular tracking, magnetic separation technologies (e.g., rapid DNA sequencing) early detection of inflammatory, cancer, diabetes, and atherosclerosis.[11–20] All these biomedical applications require that the nanoparticles have high magnetization values so as to provide high-resolution MR images. In general, the superparamagnetic nanoparticles resemble excellent imaging probes to be used as MRI contrast agents since the MR signal intensity is significantly modulated without any compromise in its *in vivo* stability.[21] Basically, all contrast agents induce a decrease in the T1 and T2 relaxation times of surrounding water protons and thereby manipulate the signal intensity of the imaged tissue.[22]

Converging advances in the understanding of the molecular biology of various diseases recommended the need of homogeneous and targeted imaging probes along with a narrow size distribution in between 10 and 250 nm in diameter. Developing magnetic nanoparticles in this diameter range is a complex process and various chemical routes for their synthesis have been proposed. These methods include microemulsions, sol–gel syntheses, sonochemical reactions, hydrothermal reactions, hydrolysis and thermolysis of precursors, flow injection syntheses, and electrospray syntheses.[23–29] However, the most common method for the production of magnetite nanoparticles is the chemical coprecipitation technique of iron salts.[30–34] The main advantage of the coprecipitation process is that a large amount of nanoparticles can be synthesized but with limited control on size distribution. This is mainly due to that the kinetic factors are controlling the growth of the crystal. Thus the particulate magnetic contrast agents synthesized using these methods include ultrasmall particles of iron oxide (USPIO) (10–40 nm), small particles of iron oxide (SPIO) (60–150 nm). Besides, monocrystalline USPIOs are also called as monocrystalline iron oxide nanoparticles (MIONs), whereas MIONs when cross-linked with dextran they are called crosslinked iron oxide nanoparticles CLIO (10–30 nm).[35–37] The modification of the dextran coating by carboxylation leads to a shorter clearance half-life in blood.[38] Hence, ferumoxytol (AMAG Pharmaceuticals), a carboxyalkylated polysaccharide coated iron oxide nanoparticle, is already described as a good first-pass contrast agent but uptake by macrophages is unspecific and too fast to enhance the uptake in macrophage-rich plaques.

**Figure 1:**
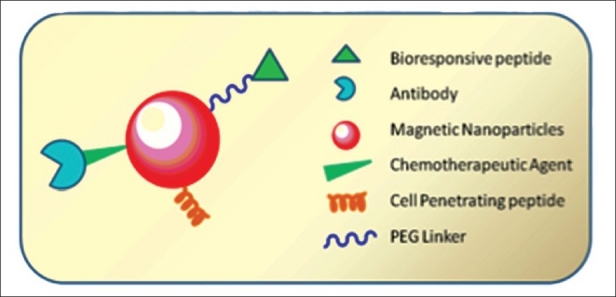
Schematic diagram representing the fucntionalization of magnetic nanoparticles with bioresponsive peptide, PEG linker, chemotherapeutic agent, antibody, and cell-penetrating peptide.[11]

In order to improve the cellular uptake, these particles can be modified with a peculiar surface coating so that they can be easily conjugated to drugs, proteins, enzymes, antibodies, or nucleotides and can be directed to an organ, tissue, or tumor [Figure 1].[7,39] While traditional contrast agents distribute rather nonspecifically, targeted molecular imaging probes based on iron oxide nanoparticles have been developed that specifically target body tissue or cells.[7,40] For instance, Conroy and coworkers developed (chlorotoxin (CTX)) a biocompatible iron oxide nanoprobe coated with poly(ethylene glycol) (PEG), which is capable of specifically targeting glioma tumors *via* the surface-bound targeting peptide.[41] Further, MRI studies showed the preferential accumulation of the nanoprobe within gliomas. In another study, Apopa *et al*. engineered iron oxide nanoparticles that can induce an increase in cell permeability through the production of reactive oxygen species (ROS) and the stabilization of microtubules.[42] These are the few applications of iron oxide nanoparticles in biomedical imaging.

These studies provide a new insight into the bioreactivity of engineered iron nanoparticles, which can provide potential applications in medical imaging or drug delivery. The further development and modification of the complexes of iron oxide along with dendrimers, polymeric nanoparticles, liposomes, and solid lipid nanoparticles are widely studied. However, the toxicity of these magnetic nanoparticles to certain types of neuronal cells is still the matter of concern.[43]

## Gold Nanoparticles

**Figure 2:**
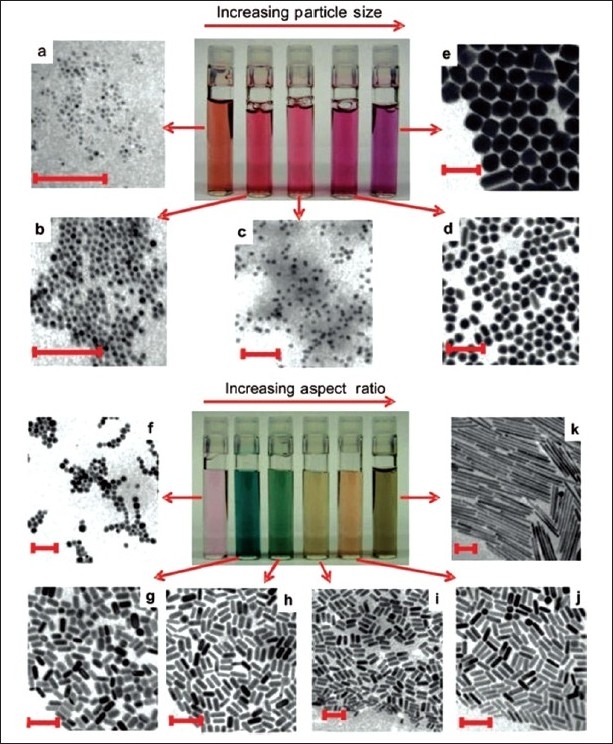
Photographs of aqueous solutions of gold nanospheres (upper panels) and gold nanorods (lower panels) as a function of increasing dimensions. Corresponding transmission electron microscopy images of the nanoparticles are shown (all scale bars 100 nm). The difference in color of the particle solutions is more dramatic for rods than for spheres. This is due to the nature of plasmon bands (one for spheres and two for rods) that are more sensitive to size for rods compared with spheres. For spheres, the size varies from 4 to 40 nm (TEMs a-e), whereas for rods, the aspect ratio varies from 1.3 to 5 for short rods (TEMs f-j) and 20 (TEM k) for long rods[50]

**Figure 3:**
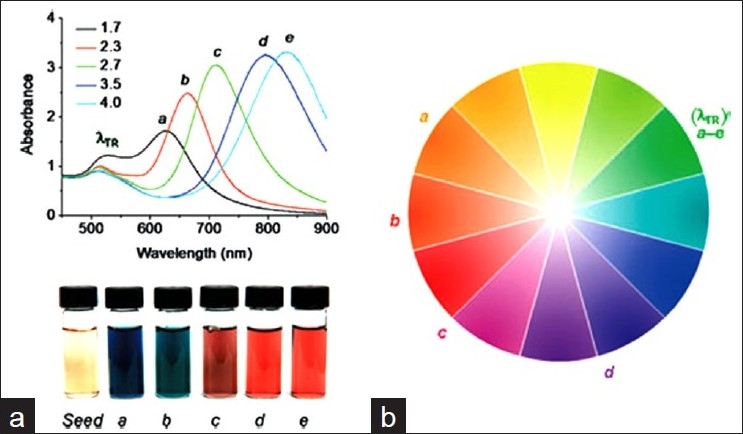
Gold nanorods (NRs) with tunable optical absorptions at visible and near-infrared wavelengths; a) Optical absorption spectra of gold NRs with different aspect ratios (a–e); b) Color wheel, with reference to gold NRs labeled a–e. TR, transverse resonance[51]

Colloidal gold, also known as gold nanoparticles, is a suspension (or colloid) of nanometer-sized particles of gold. The history of these colloidal solutions dates back to Roman times when they were used to stain glass for decorative purposes.[44] However, the modern scientific evaluation of colloidal gold did not begin until Michael Faraday’s work of the 1850s, when he observed that the colloidal gold solutions have properties that differ from the bulk gold.[45,46] Hence the colloidal solution is either an intense red color (for particles less than 100 nm) or a dirty yellowish color (for larger particles) as shown in Figures 2 and 3.[47,48] These interesting optical properties of these gold nanoparticles are due to their unique interaction with light.[49] In the presence of the oscillating electromagnetic field of the light, the free electrons of the metal nanoparticles undergo an oscillation with respect to the metal lattice.[50–53] This process is resonant at a particular frequency of the light and is termed the localized surface plasmon resonance (LSPR). After absorption, the surface plasmon decays radiatively resulting in light scattering or nonradiatively by converting the absorbed light into heat. Thus for gold nanospheres with particle size around 10 nm in diameter have a strong absorption maximum around 520 nm in aqueous solution due to their LSPR. These nanoshperes show a stokes shift with an increase in the nanosphere size due to the electromagnetic retardation in larger particles.

Moreover, the properties and applications of colloidal gold nanoparticles also depend upon its shape. Figure 2 shows that the difference in color of the particle solutions is more dramatic for rods than for spheres. For example, the rod-shaped nanoparticles have two resonances: one due to plasmon oscillation along the nanorod short axis and another due to plasmon oscillation along the long axis, which depends strongly on the nanorod aspect ratio, that is, length-to-width ratio.[54,55] When the nanorod aspect ratio is increased, the long-axis LSPR wavelength position red shifts from the visible to the NIR and also progressively increases in oscillator strength [Figure 2].[55] For example, rodlike particles have both transverse and longitudinal absorption peak, and anisotropy of the shape affects their self-assembly.[56] Due to these unique optical properties, gold nanoparticles are the subject of substantial research, with enormous applications including biological imaging, electronics, and materials science.[57] Thus to develop gold nanoparticles for specific applications, reliable and high-yielding methods including those with spherical and nonspherical shapes have been developed over the period of years.[56,58]

The most prevalent method for the synthesis of monodisperse spherical gold nanoparticles was pioneered by Turkevich *et al*. in 1951 and later refined by Frens *et al*. in 1973.[59–62] This method uses the chemical reduction of gold salts such as hydrogen tetrachloroaurate (HAuCl_4_) using citrate as the reducing agent. This method produces monodisperse spherical gold nanoparticles in the range of 10–20 nm in diameter. However, the synthesis of larger gold nanoparticles with diameters between 30 and 100 nm was reported by Brown and Natan *via* seeding of Au^3+^ by hydroxylamine.[63] Subsequent research led to the modification of the shape of these gold nanoparticles resulting in rod, triangular, polygonal rods, and spherical particles.[64–66] These ensuing gold nanoparticles have unique properties, providing a high surface area to volume ratio. Moreover, the gold surface offers a unique opportunity to conjugate ligands such as oligonucleotides, proteins, and antibodies containing functional groups such as thiols, mercaptans, phosphines, and amines, which demonstrates a strong affinity for gold surface.[67] The realization of such gold nanoconjugates coupled with strongly enhanced LSPR gold nanoparticles have found applications in simpler but much powerful imaging techniques such as dark-field imaging, SERS, and optical imaging for the diagnosis of various disease states.[68]

**Figure 4:**
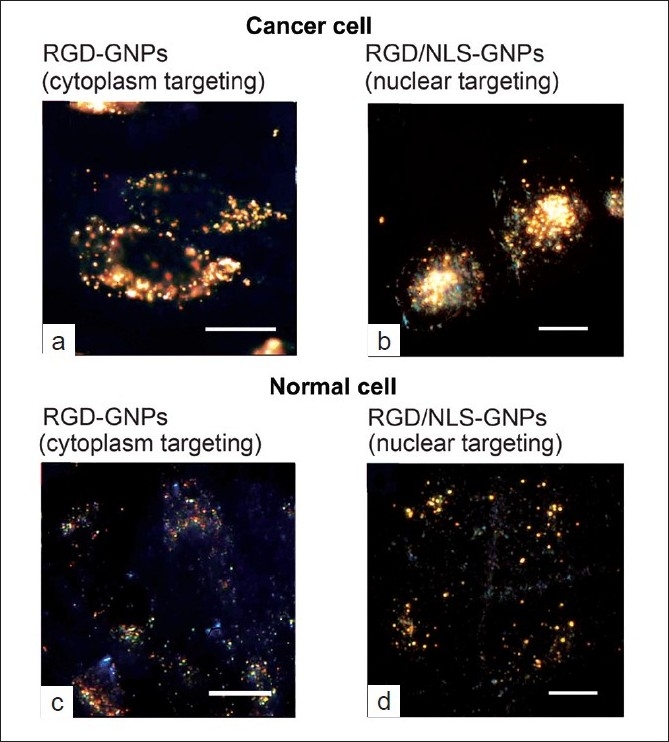
Dark field light scattering images of cytoplasm and nuclear targeting AuNPs. a) RGD-AuNPs located in the cytoplasm of cancer cells. b) RGD/NLS-AuNPs located at the nucleus of cancer cells. c) RGD-AuNPs located in the cytoplasm of normal cells. d) RGD/NLS-AuNPs located at the nucleus of normal cells. The cancer and normal cells were incubated in the presence of these AuNPs at a concentration of 0.4 nM for 24 hours and these images clearly display the efficient uptake of AuNPs in cancer cells compared with normal cells. Scale bar 10 μm[72]

In fact, El Sayed *et al*. have established the use of gold nanoparticles for cancer imaging by selectively transporting AuNPs into the cancer cell nucleus. In order to selectively transport the AuNPs into the cancer cell nucleus, they conjugated arginine–glycine–aspartic acid peptide (RGD) and a nuclear localization signal peptide (NLS) to a 30-nm AuNPs *via* PEG.[69] RGD is known to target α_v_β_6_ integrins receptors on the surface of the cell, whereas NLS sequence lysine–lysine–lysine–arginine–lysine (KKKRK) sequence is known to associate with karyopherins (importins) in the cytoplasm, which enables the translocation to the nucleus.[70–72] Thus the presence of RGD will enable cancer-cell-specific targeting, whereas the presence of NLS will exhibit cancer cell nucleus specific targeting. This intuitively developed particle was then targeted to human oral squamous cell carcinoma (HSC) having α_v_β_6_ integrins overexpressed on the cell surface (cancer model), and human keratinocytes (HaCat) (control). The authors further demonstrated that RGD-AuNPs specifically target the cytoplasm of cancer cells [Figure 4a] over that of normal cells [Figure 4c], and the RGD/NLS-AuNPs specifically target the nuclei of cancer cells [Figure 4b] over those of normal cells [Figure 4d].[69]

Similarly, Qian *et al*. reported the development of tumor-targeted gold nanoparticles as a probe for Raman scatters *in vivo*.[73] These gold nanoparticles were encoded with a Raman reporter and further encapsulated with a thiol-modified PEG coat. Additionally, to specifically target tumor cells, the pegylated gold nanoparticles were then conjugated with an antibody against epidermal growth factor receptor, which is sometimes overexpressed in certain types of cancer cells. The Raman enhancement from these tailored particles was then observed with electronic transitions at 633 or 785 nm *via* SERS. The results obtained by Qian and coworkers suggest the highly specific recognition and detection of human cancer cells, as well as active targeting of EGFR-positive tumor xenografts in animal models can be made using SERS.[73]

Moreover, the use of gold nanorods as photothermal agents sets them apart from all nanoprobes. Photothermal therapy (PTT) is a procedure in which a photosensitizer is excited with specific band light (mainly IR). This activation brings the sensitizer to an excited state where it then releases vibrational energy in the form of heat. The heat is the actual method of therapy that kills the targeted cells. One of the biggest recent successes in photothermal therapy is the use of gold nanoparticles. Spherical gold nanoparticles absorptions have not been optimal for *in vivo* applications. This is because the peak absorptions have been limited to 520 nm for 10 nm diameter. Moreover, skin, tissues, and hemoglobin have a transmission window from 650 up to 900 nm. This was circumvented by the recent invention of gold nanorods by Murphy and Coworkers, who were able to tune the absorption peak of these nanoparticles, which can also be tuned from 550 nm up to 1 *μ*m just by altering its aspect ratio of the nanorods [Figure 3].[48,74] Hence, for the rod-shaped gold nanoparticles with the absorption in the IR region, when selectively accumulated in tumors when bathed in laser light (in the IR region), the surrounding tissue is barely warmed, but the nanorods convert light to heat, killing the malignant cells. This potential application of gold nanorods sanctifies them from other nanoprobes. However, their incompatibility with other high-resolution imaging techniques such as MRI and irreproducibility in shapes led to the invention of nanocages and nanoshells.

## Nanoshells and Nanocages

**Figure 5:**
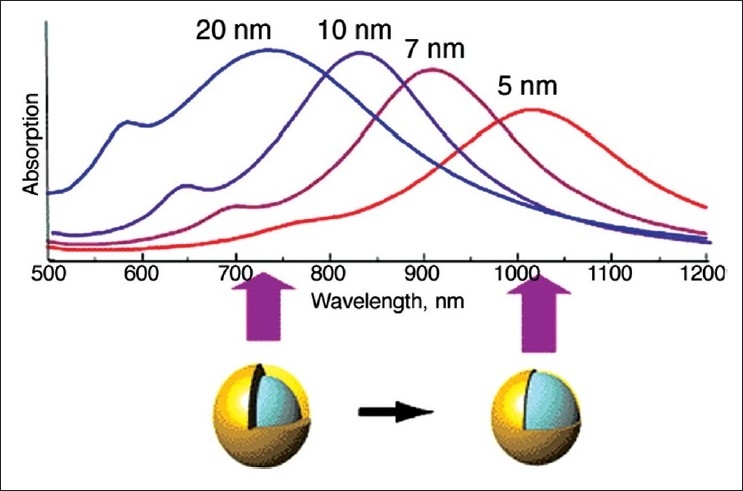
Gold nanoshell plasmon resonances for a 120-nm core with indicated shell thickness[81]

**Figure 6:**
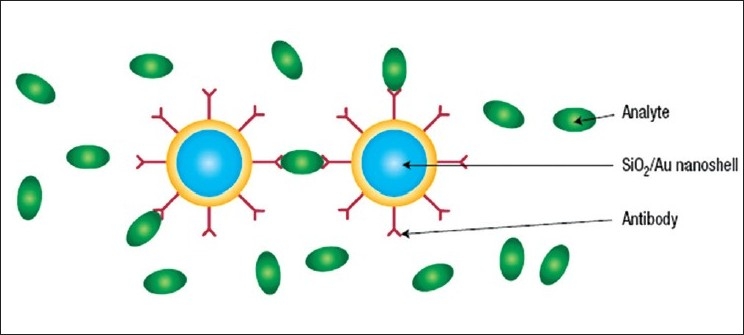
Formation of nanoshell dimer with the interaction of antibodies immobilized on the surface of the nanoshells[82]

Neeves and Birnboim calculated that a composite spherical particle consisting of a metallic shell and a dielectric core could give rise to LSPR modes with their wavelengths tunable over a broad range of the electromagnetic spectrum.[75] Later on, the experimental and theoretical work by Naomi Halas and Peter Nordlander at Rice University showed that the resonance of a silica-gold nanoshell particle can easily be positioned in the near-infrared (800–1,300 nm) region, where absorption by biomatters is low [Figure 5].[76–78] They developed silica-gold nanospheres by using freshly formed amine-terminated silica spheres. These amine terminated silica spheres were then treated with a suspension of gold colloids (1–2 nm in size). Gold was deposited *via* chemical reduction to cover the silica core and to the amine terminal of the silicon core. Although this method is widely used, the intricacy involved in the control of thickness and smoothness of the metallic shells makes this method unsuitable for the routine synthesis of controlled particle-sized nanoshells. Furthermore, they also showed the successful irreversible photothermal ablation of tumor tissue both in vitro and in vivo when these nanoshells were localized onto the tumor cells. In another study, Halas and West established the use of near-infrared resonant nanoshells for whole-blood immunoassays. They further showed that the nanoshells when conjugated with antibodies act as recognition sites for a specific analyte [Figure 6]. The analyte causes the formation of dimmers, which will modify the LSPR.[79] Subsequent work in this field led to the development of the multifunctional magnetic gold nanoshells (Mag-GNS) by Jaeyun *et al*. utilizing Fe_3_O_4_ nanoparticles as the magnetic core. The Fe_3_O_4_ nanoparticles allow MRI for diagnosis, and the gold nanoshells enable photothermal therapy. By attaching an antibody to the Mag-GNS by a PEG linker, cancer cells can be targeted. Once localized, these particles enable the detection of cancer using MRI, whereas the photothermal therapy can be used to get rid of cancer cells.[80]

Similar to gold nanoshells, gold nanocages represent a novel class of nanostructures that are hollow porous gold nanoparticles that absorb light in the near-infrared range. They were first developed by the Xia and Coworkers *via* the reaction of silver nanoparticles with chloroauric acid (HAuCI_4_) in boiling water.[81] Their LSPR peaks can also be tuned to the near infrared region by controlling the thickness and porosity of the walls. Comparable to nanoshells they have found applications in drug delivery and/or controlled drug release. Furthermore, the hollow interiors can host small objects such as magnetic nanoparticles to construct multifunctional hybrid nanostructures diagnostic imaging and therapy.

## Silver Nanoparticles

Silver nanoparticles are the particles of silver, with particle size between 1 and 100 nm in size. While frequently described as being “silver” some are composed of a large percentage of silver oxide due to their large ratio of surface to bulk silver atoms. Like gold nanoparticles, ionic silver has a long history and was initially used to stain the glass for yellow. Currently, there is also an effort to incorporate silver nanoparticles into a wide range of medical devices, including bone cement, surgical instruments, surgical masks, etc. Moreover, it has also been shown that ionic silver, in the right quantities, is suitable in treating wounds.[82–84] In fact, silver nanoparticles are now replacing silver sulfadiazine as an effective agent in the treatment of wounds. Additionally, Samsung has created and marketed a material called Silver Nano, which includes silver nanoparticles on the surfaces of household appliances. Moreover, due to their attractive physiochemical properties these nanomaterials have received considerable attention in biomedical imaging using SERS. In fact, the surface plasmon resonance and large effective scattering cross-section of individual silver nanoparticles make them ideal candidates for molecular labeling.[85] Thus many targeted silver oxide nanoprobes are currently being developed.

Typically, they are synthesized by the reduction of a silver salt with a reducing agent like sodium borohydride in the presence of a colloidal stabilizer. The most common colloidal stabilizers used are polyvinyl alcohol, poly(vinylpyrrolidone), bovine serum albumin (BSA), citrate, and cellulose. Newer novel methods include the use of β-d-glucose as a reducing sugar and a starch as the stabilizer to develop silver nanoparticles ion implantation used to create silver nanoparticles.[86] Also, it is important to note that not all nanoparticles created are equal. The size and shape have been shown to have an impact on its efficacy. In fact, Elechiguerra *et al*. demonstrated that silver nanoparticles undergo a size-dependent interaction with HIV-1, with particles exclusively in the range of 1–10 nm attached to the virus.[87] They further suggest that silver nanoparticles interact with the HIV-1 virus via preferential binding to the gp120 glycoprotein knobs.[87] Similarly, Furno and Coworkers have developed biomaterials by impregnating silicone coated with silver oxide nanoparticles using supercritical carbon dioxide.[88] These novel biomaterials were developed with an aim to reduce the antibacterial infection. The results obtained were mixed but the methodology allows for the first-time silver impregnation (as opposed to coating) of medical polymers and promises to lead to an antimicrobial biomaterial.[88]

Even though these particles are not as widely preferred as compared to the gold nanoparticles and nanoshells, but they have made a tremendous impact on today’s era of medical science. The interesting property of the noble metals is a promise that they would be continuously used as newer applications and protocols are being developed.

## Conclusion

This review article provides a glimpse to some simpler nanoparticles which are being currently modified for their potential applications in medicine. However, the field of nanoscience has blossomed over the last two decades and the need for nanotechnology to explore beyond the cells walls has become more important.[89] Nanoparticles have successfully come to aid various disease states, but the advances in biomedical imaging depend largely on the shape, size, and selectivity of the nanoparticle to the target.[89] Moreover, the type of the particle synthesized also governs the imaging modality to be used and thus the cost of diagnosis. Even though current investigations have demonstrated that multivalent composite materials can provide significant advantages, the ambiguity in developing them for a particular target with high specificity is still challenging. Fortunately, the field of nanotechnology continues to grow interest within the chemical research community with major discoveries as well as new scientific challenges.[44] Nevertheless, the future studies should also aim to address safety and biocompatibility of these nanoparticles, in particular long-term toxicities.[90] Additional clinical studies on humans and on animal models should be performed to substantiate their use especially in biomedical imaging using MRI, CT, ultrasound, PET, SERS, and optical imaging.[90]
